# Host Range of the Mikrocytid Parasite *Paramikrocytos canceri* in Decapod Crustaceans

**DOI:** 10.3390/pathogens8040252

**Published:** 2019-11-20

**Authors:** Morgan Edwards, Christopher J. Coates, Andrew F. Rowley

**Affiliations:** Department of Biosciences, College of Science, Swansea University, Swansea SA2 8PP, Wales, UK; morgan_edwards123@hotmail.com

**Keywords:** Mikrocytid, antennal gland, histopathology, fisheries, *Cancer pagurus*, disease connectivity

## Abstract

Mikrocytids are a widespread but rather neglected group of parasites of aquatic invertebrates. One such parasite is *Paramikrocytos canceri*—discovered to infect the antennal gland of the juvenile edible crab, *Cancer pagurus*, taken from several intertidal sites across the United Kingdom. To determine if this parasite is also present in other species of decapod crustaceans, we surveyed crabs (*n* = 330) across two contrasting sites in Pembrokeshire (UK). Using a histopathological approach, *P. canceri* infection was confirmed in variable numbers of edible crabs from both survey sites, 7–44%. No measurable signs of infection were encountered in four other co-located species, including European shore crabs (*Carcinus maenas*), Montagu’s crabs (*Xantho hydrophilus*), velvet swimming crabs (*Necora puber*) and broad-clawed porcelain crabs (*Porcellana platycheles*). These data imply that *P. canceri* has a more limited host range than suggested by molecular diagnosis alone.

## 1. Introduction

The edible (brown) crab, *Cancer pagurus*, is an economically sensitive species of decapod crustacean in Northern Europe and valued at ~£50 million annually to Ireland and the UK [1]. Due to this economic importance, it has been subject to several studies on its disease susceptibility [2,3,4,5]. Edible crabs are prone to infection by a wide range of micro- and macro-parasites, some of which are considered to have significant consequences to fishery outputs [6]. For example, juvenile *C. pagurus* are highly susceptible to parasitization by the dinoflagellate, *Hematodinium* sp. [3], with high levels of infection up to 30% reported in one location within the Bristol Channel region of the UK [7]. A further potentially serious infection of juvenile (pre-recruit) edible crabs is caused by members of a novel group of parasites belonging to the proposed order Mikrocytida [8,9,10], which share some similarities with their “sister” group, the Haplosporida [10]. Indeed, the initial reports on the presence of such parasites from edible crabs collected in the English Channel and Pembrokeshire, South West Wales, UK misidentified these as “haplosporidian-like” [5,11]. Pathology symptoms manifest as extensive hypertrophy of the antennal gland (visible upon dissection). Histological examination of such tissues reveals a range of severity from light infections with small numbers of parasites in the epithelial cells of the antennal gland through to high-grade infections with hypertrophic cells filled with uni- and multicellular forms of these parasites. Various morphotypes of parasites appear in the lumen of the antennal gland, and in this late phase of infection, they are found in the epithelial cells underlying the cuticle and in the nephrocytes of the gills. Presumably, the spread from the antennal gland to gills is brought about when parasites spill into the haemolymph (described in [11]). There remains a lack of information about whether this disease alone causes mortality in crabs.

Due to the expanded use of next generation sequencing, it was confirmed that the parasite is not a haplosporidian but another form of mikrocytid, *Paramikrocytos canceri* [10]. This group of parasites has received relatively little attention with the exception of *Mikrocytos mackini*—that is the causative agent of Denman Island disease in the Pacific oyster, *Crassostrea gigas* [12]. *Mikrocytos*-like parasites have been isolated from Manila clams, *Ruditapes philippinarum,* in northwest Spain [13], and more recently, two novel species (*M. veneroïdes* and *M. donaxi*) were found to be involved in mass mortality events of the wedge clam, *Donax trunculus,* in France [14]. The discovery of an infectious mikrocytid in edible crabs (i.e., *P. canceri*) is particularly exciting as the authors [10] were able to find molecular signatures of its presence in a wide range of aquatic invertebrates that could suggest either an extensive host range or the existence of many vectors for infection. Furthermore, the observation that other decapods co-inhabiting the intertidal zone with juvenile edible crabs, such as the European shore crab (*Carcinus maenas*), may also harbour this parasite and other noxious agents [15], is important from a fisheries management perspective.

Herein, our aim was to investigate whether other decapod crustaceans from the same intertidal location act as co-hosts for these potentially dangerous parasites. To this end, we performed a histopathological survey of 330 crabs comprised of five species across two sites, Freshwater East and Pembroke Ferry (Pembrokeshire, UK), shown previously to harbour parasitized (*P. canceri*) edible crabs [11,16]. This study forms part of a wide baseline survey of diseases of on-shore, juvenile edible crabs from the Irish and Celtic Seas (reviewed by [17]).

## 2. Results and Discussion 

A total of 330 crabs were collected over the months of April and June 2013, 157 from Freshwater East and 173 from Pembroke Ferry (Table 1). Three species of crabs were consistently found at the two sites, namely broad-clawed porcelain crabs (*Porcellana platycheles*)*,* edible (brown) crabs (*C. pagurus*) and European shore crabs (*Carcinus maenas*). Other crabs, including the Montagu’s crab (*Xantho hydrophilus*) and velvet swimming crabs (*Necora puber*)*,* were collected in similar numbers from Freshwater East only.

**Figure 1:**
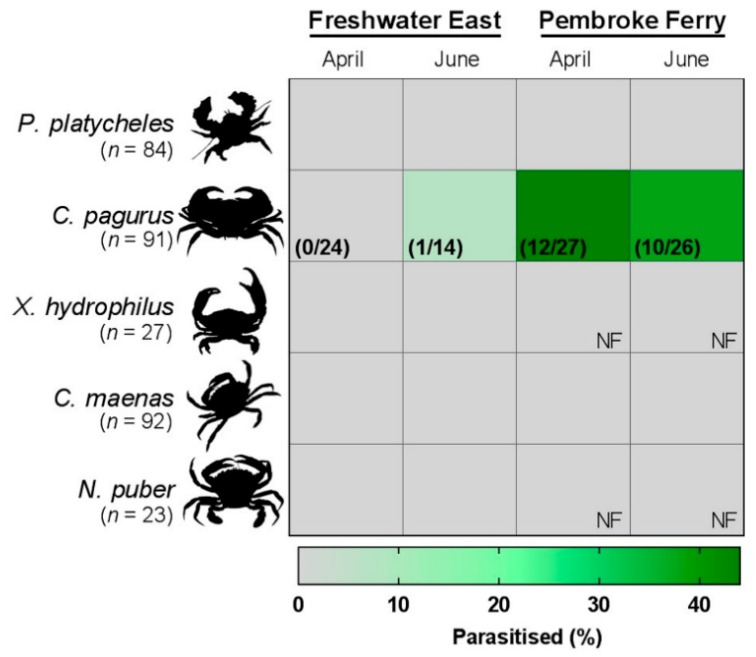
Prevalence (%) of a mikrocytid infection of the antennal gland of juvenile *Cancer pagurus*. Crabs were dissected and assessed histologically for symptoms of *Paramikrocytos canceri*. Grey represents no measurable signs of infection in the crabs surveyed. NF – indicates “none found” at the respective site.

Parasitism of edible crabs by this mikrocytid was confirmed at both sites. The proportion of infected animals at Pembroke Ferry was significantly higher (38% difference, *p* < 0.001) than at Freshwater East (Figure 1), unlike earlier reports from the same survey sites where the prevalence of infection was not significantly different [11,16]. Evaluation of our diagnostic test revealed a high sensitivity value of 0.974 (95%CI: 0.865 – 0.999)—i.e., diseased animals were correctly identified as positive.

**Figure 2:**
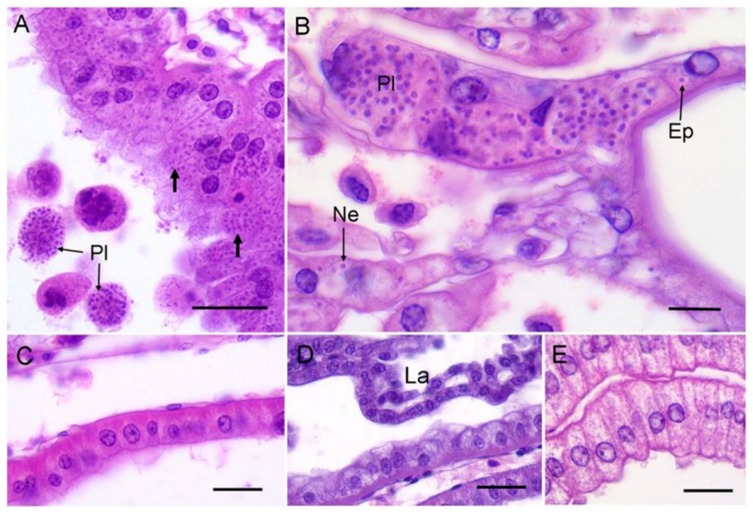
(**A**) Example of mikrocytid infection of the antennal gland from an edible crab, *C. pagurus*. Note both unicellular (unlabelled arrows) and free plasmodial (Pl) forms of the parasite. Scale bar = 25 µm. (**B**) High power micrograph of gill from an edible crab showing plasmodia (Pl) and unicellular forms of the mikrocytid parasites within epithelial cells (Ep) underlying the cuticle, and in nephrocytes (Ne). Scale bar = 10 µm. (**C**) Example of an uninfected antennal gland of a shore crab, *C. maenas*, overlying the hepatopancreas. Scale bar = 25 µm. (**D**) Anterior labyrinth (La) region of the antennal gland from an uninfected shore crab. Scale bar = 25 µm. (**E**) Uninfected antennal gland cells from a Montagu crab, *X. hydrophilus*. Scale bar = 25 µm.

The antennal gland of infected crabs was hypertrophic with a distinct shiny, yellow appearance (previously established as a diagnostic tissue aberration [11]). Histologically, the compromised antennal gland showed marked hypertrophy and the presence of intracellular unicellular microcells and extracellular plasmodia (Figure 2A). In severe cases, the infection spreads to the epithelial cells and nephrocytes of the gills (Figure 2B). Free unicellular forms and plasmodia could also be found circulating in the haemolymph of grossly infected crabs (severity Level 4 according to Thrupp et al. (2013) [11]).

None of the other species of crabs examined by gross dissection or by tissue-specific histology showed evidence of infection in the antennal gland, haemolymph or gills by this mikrocytid parasite (Figure 2C–E). Our data imply that at least at these two sites and in the two months sampled, the other species of crabs do not appear to be susceptible to infection by *P. canceri*. The detection of the mikrocytid parasite by Hartikainen et al. (2014) [10] using a highly specific PCR assay on shore crabs (*C. maenas*) is most likely due to association of the parasites with these animals without any true parasitism. Alternatively, the putative *P. canceri* infection cycle in shore crabs could be housed in tissues other than those associated traditionally with this group of parasites. Application of sensitive (q)PCR-based methodologies are important for monitoring the presence/absence of notifiable (regulated) disease-causing agents such as mikrocytids in environmental samples and putative hosts (e.g., [14,18,19]), but do not necessarily reflect host–pathogen antibiosis. Whatever the case, the host range and distribution of these novel parasites need to be fully investigated in order to gauge the risk to commercial shellfish stocks (crustacean and bivalve) in the Irish and Celtic Seas.

## 3. Materials and Methods 

### 3.1. Survey Sites

Crabs were collected from two intertidal sites in Pembrokeshire, UK. The first site was at Freshwater East (51°39′0’’ N, 4°52′0″ W; Grid Reference SS16984), a rocky shore facing the Bristol Channel. The second was at Pembroke Ferry (51°42′15″ N, 4°55′59″ W; Grid Reference SM974046) located in the Daugleddau Estuary at the head of the Milford Haven Waterway. This area experiences sea traffic year-round and is a site close to oil refineries fed by tankers. Crabs were collected at the best low tides in April and June 2013 and transported back to the university in damp seaweed.

### 3.2. Laboratory Procedures

Each crab was given an individual ID code, sexed, the dorsal and ventral surfaces photographed, and carapace width (CW) measured. Any notable conditions including missing or damaged limbs, the presence of exoskeletal abnormalities, such as shell disease and moult status were also recorded. All crabs were prepared for histology using the methods described in Thrupp et al. (2013, 2015) [11,16]. Briefly, small crabs (generally <25 mm CW) were fixed whole following the injection of Davidson’s sea water fixative, rinsed in water, bisected and post-fixed in a decalcifying solution (4% formalin, 5% EDTA) for 4–5 days. Larger crabs (>25 mm CW) were injected with Davidson’s sea water fixative, and the gills and hepatopancreas with attached antennal gland removed and post-fixed for ca. 24 hours in further Davidson’s sea water fixative. Sections were cut at ca. 7 µm, stained with Cole’s haematoxylin and eosin, examined and photographed using an Olympus BX41 microscope with an Olympus SC30 camera.

### 3.3. Data Handling

The antennal gland was located successfully from 91 out of the 104 edible crabs collected. Contingency (proportional) analysis using Fisher’s exact test was applied to parasite incidence(s) data across both sampling sites/months, with Wilson–Brown’s method used to calculate the confidence intervals for the sensitivity of our diagnostic test. Data were visualised and analysed in GraphPad PRISM v7.

## Figures and Tables

**Table 1:** General features of crabs collected from Freshwater East and Pembroke Ferry.

Common Name *[Scientific Name]*	Freshwater East					Pembroke Ferry				
	N	Mean CW (mm)	Size Range of CW (mm)	Sex Ratio (M:F)	Status (Juvenile: Adult) *	N	Mean CW (mm)	Size Range of CW (mm)	Sex Ratio (M:F)	Status (Juvenile: Adult) *
Broad clawed porcelain crab *[Porcellana platycheles]*	30	8	4–10	1:2	12:18	54	8	4–12	1:3.6	21:33
Edible (brown) crab *[Cancer pagurus]*	45	31	10–77	1:0.2	45:0	59	38	14–76	1:0.4	59:0
Montagu’s crab *[Xantho hydrophilus]*	27	39	21–55	1:0.7	10:13	-	-	-	-	-
Shore crab *[Carcinus maenas]*	32	27	9–57	1:7	28:4	60	23	8–65	1:29	57:3
Velvet swimming crab *[Necora puber]*	23	27	14–53	1:7	10:13	-	-	-	-	-

Note: * Sex determined by gross examination at dissection and/or by histology. CW: carapace width.
